# Socioeconomic profile of couples seeking the public healthcare system (SUS) for infertility treatment

**DOI:** 10.5935/1518-0557.20160026

**Published:** 2016

**Authors:** Rachel Tavares, Gisele Cunha, Lilian Aguiar, Shaytner Campos Duarte, Nilza Cardinot, Elizabeth Bastos, Francisco Coelho

**Affiliations:** 1 Centro de Infertilidade e Medicina Fetal do Norte Fluminense / Departamento de Medicina Reprodutiva do Hospital Escola Alvaro Alvim. Campos dos Goytacazes/RJ, Brazil; 2 Faculdade de Medicina de Campos dos Goytacazes. Campos dos Goytacazes/RJ, Brazil; 3 Prefeitura Municipal de Campos dos Goytacazes. Campos dos Goytacazes/RJ, Brazil

**Keywords:** Assisted human reproduction, Infertility, Socioeconomic status

## Abstract

**Objective:**

The number of couples seeking assisted reproduction services in pursuit of the dream of conceiving a child is growing. In developing countries 10 to 15% of couples of childbearing age cannot bear a child by natural means and the impossibility of conceiving a child has a significant impact on the health and well-being of the couple. The aim of this study was to evaluate the socioeconomic profile and the main causes of infertility of couples seeking assisted reproduction treatment through the public healthcare system.

**Methods:**

We analyzed 600 medical records of couples who sought infertility treatment at the public healthcare system, and we divided them into three groups according to age: 35 years, 35 to 39, and 40 years or more. In each group we analyzed the cause of infertility, the number of children of the spouses, the education level and family income.

**Results:**

The main cause of infertility was male-related in 34%, followed by tubal factor in 31.5%. We found that 56% of the women were less than 35 years old and 58% of the couples earned less than 3 minimum wages.

**Conclusion:**

The profile of the couples was: low-income, low education and less than 35 years of age. The cost of assisted reproductive treatment is still high, being restricted to couples of higher socioeconomic statuses. An effective public healthcare policy could minimize this problem by improving the quality of care for couples seeking infertility treatment at the public healthcare system.

## INTRODUCTION

With the spread of assisted human reproduction techniques throughout the world, the number of infertile couples seeking assisted reproduction services for conceiving a child is increasing (Fideler& Bernstein, 1999).

The World Health Organization (WHO) estimates that in developing countries there are 10 to 15% of couples of childbearing age, with an increasing incidence in relation to age, that cannot bear a child by natural methods, which means that 1 in every 7 couples is considered infertile (Mascarenhas et al, 2012; Sembuya, 2010; Chachamovich, 2010). Data from the Brazilian Institute of Geography and Statistics (IBGE) shows that, in Brazil, there are approximately 4 million couples with infertility (IBGE, 2010).

Infertility is considered a disease of the reproductive system, defined by the absence of clinical pregnancy after 12 months of regular sexual intercourse (US Department of Health and Human Services, 2014). The impossibility of conceiving a child has a significant impact on the health and well-being of the couple (Fideler& Bernstein, 1999), making it not only a problem of the private network, but a public healthcare issue now (Mascarenhas *et al*., 1990; Cui, 2010; Macaluso *et al*., 2010).

In many developed countries, the government finances human reproduction treatments, especially in-vitro fertilization. Studies show that even couples who have access to human reproduction services at the public healthcare system face socioeconomic, racial, ethnic and financial barriers to continue the treatment (Macaluso *et al*., 2010; Bitler & Schmidt, 2006; Chandra & Stephen, 2010).

In 2010, with the goal to facilitate access to human reproduction treatment, Canada began to fund up to three cycles of in-vitro fertilization per couple. Since then, there has been a change in the socioeconomic profile of couples who started treatment at the public healthcare system, increasing the number of unemployed, low income and low educational level patients. The number of couples who sought assisted reproductive treatment, through the public healthcare system, because of secondary infertility, doubled after the deployment of this healthcare policy (Togas *et al*., 2013).

Most infertility treatments performed in developing countries are carried out at private clinics, and they are generally sought by older patients, Caucasians, with low body mass index (BMI) and high socioeconomic status, when compared to those seeking treatment at the public healthcare system (*Macaluso et al*., 2010; Bitler& Schmidt, 2006; Chandra & Stephen, 2010).

There are about 106 fertility-treatment clinics registered by Sisembrio/Anvisa of which only 8 are qualified to perform human assisted reproduction procedures by the Public Healthcare System (SUS) in Brazil. (Makuch *et al*., 2011; SisEmbrio2015). Most of them are private, limiting access to treatment, and the cost is still unmatched by the financial possibilities of most of the population. In addition, public assistance programs do not always cover all the costs of assisted reproduction care (Souza, 2014). The public healthcare system should include human reproduction treatment in their healthcare programs, ensuring the access for more people.

The aim of the study was to evaluate the socioeconomic profile and the main causes of infertility affecting couples seeking assisted reproduction treatment through the public healthcare system in the city of Campos dos Goytacazes. The assessment of this data should contribute to the development of healthcare strategies and policies, in order to facilitate access to high complexity infertility treatment at the public healthcare system.

## MATERIAL AND METHODS

We ran a cross-sectional retrospective study, analyzing 600 medical records of couples who sought the Infertility and Fetal Medicine Center at the Alvaro Alvim Teaching Hospital in the city of Campos dos Goytacazes-RJ from January 2008 to July 2014.

The records containing information on the variables were included in this study. The records that did not have any information or had incomplete data on the variables studied were excluded.

The patients were divided into three groups: GI, up to 35 years of age; GII, 35 to 39 years; and GIII, aged greater than or equal to 40 years. In each group we investigated the cause of infertility, the number of children from spouses, women's educational levels, family income and the existence of previous unions.

The monthly income of the couple was based on the minimum wage value at the time of the study, equivalent to U$ 253.40 and it was divided into four groups: GI, couples with family income less than or equal to 1 minimum wage; GII, couples with family income between 2 and 3 wages; GIII, couples with family income between 4 and 5 wages; GIV couples with family income above six minimum wages. Any kind of prior marriage or stable union was considered as previous marriage.

The causes of infertility were divided into groups: tubal factor 1, patients with tubal ligation as a permanent method of contraception; tubal factor 2, patients who had any obstructive pathology of the tubes, except for tubal ligation; endometriosis, including any grade of disease, all the way to superficial endometriosis in woman with absence of clinical pregnancy after 12 months of regular sexual intercourse; ovarian factor, patients with low ovarian reserve in woman with absence of clinical pregnancy after 12 months of regular sexual intercourse, premature ovarian failure, polycystic ovaries and anovulation; uterine factor, patients with myometrium or endometrium pathologies such as polyps and submucosal fibroids, intramural fibroids with an intracavitary component or any size fibroids distorting the uterine cavity, poor Müllerian formations and adhesions; male factor, made up of patients with alterations in the semen analysis - according to World Health Organization criteria of 2010. Among patients with male-related factors, we evaluated the possible cause of infertility, such as the presence of varicocele, vasectomy, testicular trauma, and other male genitourinary tract diseases that contribute to changes in semen - such as previous history of orchitis, testicular or epididymis tumors or cryptorchidism.

There was a group of couples with unexplained infertility, established when all other causes of infertility surveyed were excluded.

Any low complexity (timed intercourse, ovulation induction and intrauterine insemination) or high complexity (in-vitro fertilization and intracytoplasmic sperm injection - ICSI) treatment for infertility held previously to the couple's entry into the service was considered as prior treatment.

To setup a database we used Microsoft Excel spreadsheet to plot the data. Responses were numbered and participants were identified by means of codes. The data was plotted and analyzed in the Minitab software, version 15.1.1.0 (2007) and presented in graphs and tables plotted on the Excel spreadsheet. Data was analyzed using descriptive statistics and presented in the frequency of occurrence of each variable, to be expressed in absolute numbers and percentage.

The study followed ethical procedures and was approved by the Ethics Committee of the School of Medicine of Campos.

## RESULTS

From the 600 analyzed records, 37 were excluded because they did not have any information or had incomplete data on the variables, and 563 were used in the study. From these, 2 patients chose to be a single parent.

**Figure 1:**
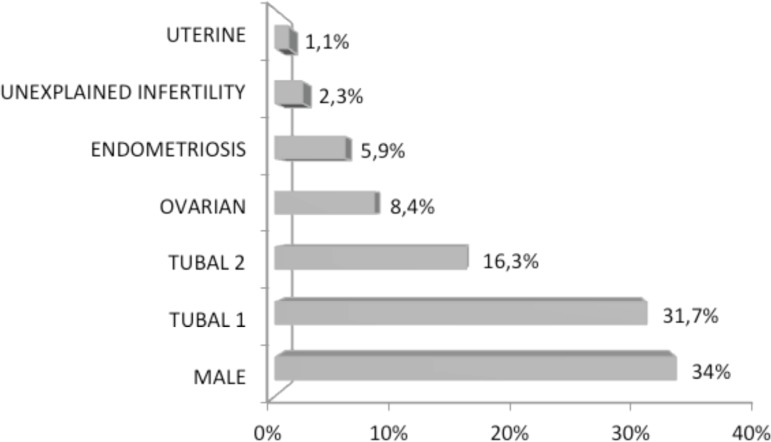
Main causes for the infertility treatment demand.

Eighty-five percent of the couples had not done been submitted to any treatment for infertility, and 15% had had some kind of low or high complexity treatment. Male factor was the cause of infertility in 34% of the couples, and among the main causes for this male factor, 76% was due to varicocele, 13% due to vasectomy, and other causes amounted to 13%. Tubal factor, due to tubal ligation was the cause of infertility in 31,7% of the couples, and other tubal disorders corresponded to 16.3% of cases. Ovarian factor, endometriosis and uterine causes accounted for 8.4%, 5.9% and 1.1%, respectively. Unexplained infertility was the cause in 2.3% of these couples (Figure 1).

**Figure 2:**
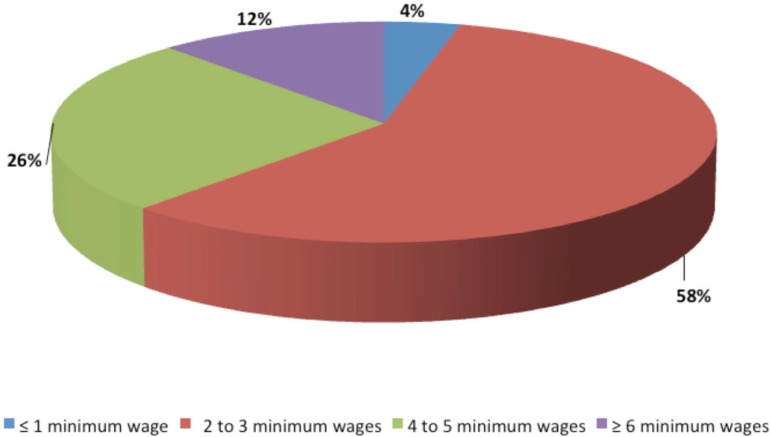
Profile of the average family income (%) of the surveyed couples.

Regarding age, 56% of infertile women were under 35 years; 27% were between 35 and 39 years of age, and 17% were 40 years old or more. Regarding family income, 4.4% of the couples earned less than 1 minimum wage, 58% had an income between 2 and 3 minimum wages, 25.8% between 4 and 5 minimum wages, and 11.7% of the couples had a monthly income higher than or equal to 6 minimum wages (Figure 2).

Regarding the group of infertile women broken down by age, it was found that those younger than 35 had the male factor as the main cause of infertility, with 43,2% of cases, the majority (61.9%) earning between 2 and 3 minimum wages; they also had completed high school (45.5%) and had no children (74%). We found that 23.4% had had previous marriage and 57.5% had not been submitted to any previous treatment for infertility. Among women 35 to 39 years old, the leading cause of infertility was tubal factor - tubal ligation, corresponding to 38.8% of cases in this age group; 52% earned between 2 and 3 minimum wages monthly; 43.4% were high school graduates; 58.6% had no children and 22.4% had two children. Of them, 38.2% had had no prior relationship and about 30% had previous treatment for infertility. Of patients over 39 years, the most common cause of infertility was tubal ligation (42.9%); over half of them (55.1%) earned between 2 and 3 wages; 41.8% had completed high school, 54.1% had no children, 22.4% had two children and 13.3%, had one child only; 45.9% had no previous union and 17.1% had not been submitted to previous infertility treatment (Table 1).

**Table 1:** Relationship between infertile women age and infertility causes.

Age	<35 y/o	35 to 39 y/o	>39 y/o
	**%**	**%**	**%**
**Causes**			
Male	43.2	27	17.3
Tubal 1	24.7	38.8	42.9
Tubal 2	16.2	17.1	15.3
Ovarian	7.5	4.6	17.3
Endometriosis	5.8	7.9	3.1
Unexplained infertility	2.3	3.3	1.0
Uterine	0.3	1.3	3.1

Regarding the number of children among the patients receiving a minimum wage or less, 76% had no children, 16% had two children and 8% three or more. Of those who earned two to three minimum wages, 62.2% had no children, 18.7% had two, 5.5% had three children and 1.2% had more than three. Between 4 to 5 minimum wages, 66.3% had no children, 17.9% had two, 9.7% and 2.1% had three and more than three children, respectively. At the six or more wages range, 81.8% had no children, 9.1% had two and 7.6% had three or more children.

Upon associating education to infertility causes and number of children, we found in patients with Junior High school degrees, the main cause of infertility was tubal factor 1, with 49.7%, followed by male factor with 23% of cases. Tubal factor 2, ovarian factor and endometriosis were 18.6%, 5.0% and 2.5 %, respectively. 43.5% had no children, 14.3% had one, 27.3% two, 11.2% three and 3.7 % more than three children. Among patients who had completed High School, the main infertility cause was the male factor (37.3%), followed by tubal factor 1 (30.9%) and tubal factor 2 (15.7%). 7.2% had an ovarian factor and 3.6% endometriosis. 7.5% had no children, 7.2 % had one, 18.5 % had two, 6.4% and 0.7 % had three and more than three children, respectively. Regarding Higher Education, the main cause of infertility was the male factor (40.1%), followed by tubal factor 2 (14.4%). Endometriosis and ovarian factor corresponded to 13.8 and 13.3%, respectively; and tubal factor 1, 13.1% of cases. 88.8 % had no children and nobody had more than three children in this group (Table 2).

**Table 2:** Relationship between causes of infertility and number of children, with education.

	Education		
	**Junior High**	**High School**	**Higher Education**
**Causes**	**(%)**	**(%)**	**(%)**
Male	43.2	27	17.3
Tubal 1	24.7	38.8	42.9
Tubal 2	16.2	17.1	15.3
Ovarian	7.5	4.6	17.3
Endometriosis	5.8	7.9	3.1
Unexplained infertility	2.3	3.3	1.0
Uterine	0.3	1.3	3.1
**Number of Children**			
0	43.5	7.5	88.8
1	14.3	7.2	3.3
2	27.3	18.5	4.6
3	11.2	6.4	3.3
>3	3.7	0.4	0

## DISCUSSION

Despite the spread of treatment methods, human reproduction is still restricted to couples with better socioeconomic level, who can afford the cost of treatment, since this remain the major impediment to reproductive treatment access (Fideler& Bernstein, 1999; Macaluso *et al*., 2010).

A Canadian study evaluated the profile of patients who received human assisted reproduction treatment before and after the country's government started to finance up to 3 cycles of in-vitro fertilization for proven infertile couples (Macaluso *et al*., 2010). Before the implementation of this healthcare policy in Canada, couples seeking infertility clinics were mostly Caucasians, with high socioeconomic level, higher educational level, higher family income, low BMI and the women were older (Macaluso *et al*., 2010). After the government policy, the profile of Canadian couples who were seeking infertility treatment changed. The women were younger, with lower family income and lower educational levels (Macaluso *et al*., 2010). The changing profile reflects the large existing financial barrier between couples and access to treatment, such observation corroborates our data regarding the profile of couples who sought access to our service.

The main infertility cause among American couples, according to a study carried out in 2014 by the United States Department of Health and Human Services, is the tubal factor followed by the male factor (US Department of Health and Human Service, 2014). The results obtained in our study reinforces the data found in the American literature, as 47.7% of couples seeking this treatment had tubal factor as a cause of infertility followed by the male factor, with 34%.

In 2012, a study carried out to estimate the trend and the prevalence of global and regional infertility since 1990, analyzed household survey data obtained through the application of demographic research and reproductive health questionnaires in several countries in Europe, Asia and Africa (Mascarenhas *et al.*, 2012). This study investigated both primary and secondary infertility.

There was little change in the prevalence of infertility between 1990 and 2010 in various regions of the world, except Africa and South Asia where there was a marked decrease during such time period (Mascarenhas *et al*., 2012). In areas where prevalence did not change much, there was an actual decrease in fertility because fewer couples tried to have children.

The overall loss of fertility in women was mainly related to old age at the time chosen to conceive a first child. They were usually women aged over 35 years with higher socioeconomic level and higher education, when compared to women with secondary infertility, who already had children and had decreased fertility over the years.

In our study, we also found that the higher the monthly income of the couple and the higher the educational level, the lower the number of children. On the other hand, couples with more children were those with lower socioeconomic status, lower family income and lower educational level, as described on table 2.

**Figure 3:**
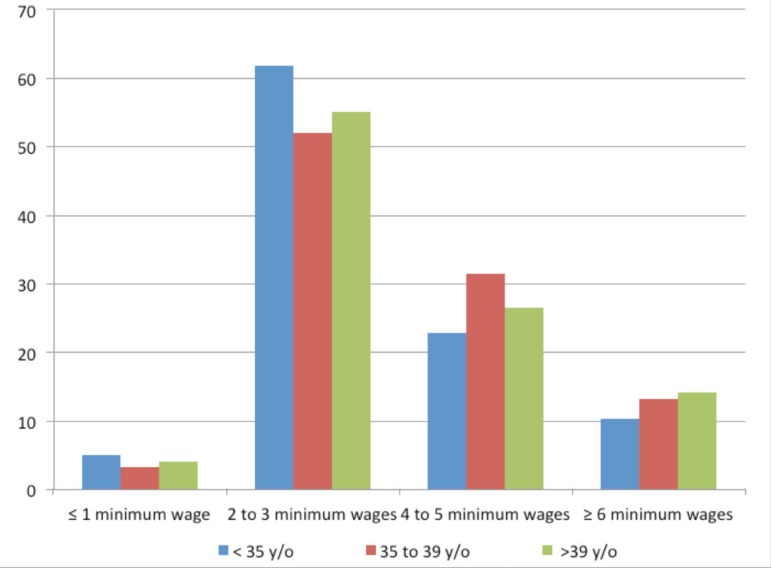
Relationship of couples seeking assisted reproduction treatment through the public healthcare system in each group of women, broken down by age (< 35; 35 to 39 years; and> 39 years) according to family income.

Still, by sorting patients by family income, we found that the difference in fertility rates was not significant among women under 35 years, 36-39 years and above 39 years of age in each group, showing that in the same socioeconomic status, the infertility rate was similar among younger and older women. Therefore, the factor that contributes to the profile of patients seeking infertility treatment at the public healthcare system is the socioeconomic status, especially the lower status, nor the age factor as it happens in the private system (Figure 3). According to Mascarenhas et al, this is so because the lower the socioeconomic status, the higher the number of children, regardless of the woman's age in these lower socioeconomic tiers, secondary infertility being predominant, mainly by tubal ligation (80%), which may reflect a policy of family planning with conflicting concepts. While at higher socioeconomic levels, the number of children is lower, and infertility occurs because of other factors, such as planning children after professional success and financial stabilization, with primary infertility as leading cause of infertility in this group (Mascarenhas et al., 2012).

Still on the educational level, the presence of 43% of childless couples in the group with junior high education may reflect the impact of this change upon behavior in the planning of families. This is supported by the observation that the number of children increases parallel to family income. This observation of reproductive behavior contrasts the outdated concept that infertility is a luxury of an elite group and the least favored population need birth control due to the number of children/family members. This data is consistent with the observations of other studies and the Brazilian reality according to IBGE surveys (2010).

In 2009 the Department of Economic and Social Affairs of the United Nations published fertility information from the world, age trends upon first conception, number of children, contraception methods and family planning (United Nations, 2011). The results of this 2009 publication were similar to data released by the WHO in 2011, linking the main cause of infertility in developing countries, including Brazil, to tubal factor, and largely due to tubal ligation, used as a contraceptive method (Mascarenhas *et al.*, 2012; United Nations, 2011; OECD, 2015).

The explanation for these results is that in developing countries, where most of the population is less socio-economically favored, women have children at a younger age and the number of children is higher, getting the final surgical sterility used as a contraceptive method in wide scale and earlier (United Nations, 2011; OECD, 2015).

Over the years, most of these women engage in new relationships and again have the desire of getting pregnant. On the other hand, in developed countries, primary infertility plays an important role, especially the older ones that plan their first pregnancy (Mascarenhas *et al*., 2012; United Nations, 2011; OECD, 2015).

This same study (United Nations, 2011) showed that the highest percentage of tubal ligation is performed in lower-income women with less education, being the leading cause of demand for infertility treatment. In contrast, endometriosis and tubal factor for other diseases of the tubes, except tubal ligation, are the main causes of infertility in women with higher socioeconomic status (United Nations, 2011; OECD, 2015). These results were similar to those found in our study.

Our results also reflect this reality, although there is a behavioral change in trend in the population, in terms of family planning these concepts are active.

In Brazil there are 106 fertility clinics registered by Sisembrio/Anvisa, of which only 8 are certified to perform human assisted reproduction procedures by the Public Healthcare System (SUS) (Makuch *et al*., 2011; SisEmbrio 2015). The waiting time for infertile couples to start treatment at the public services is usually long, causing some services to often having to stop servicing new couples in order to reduce the wait of those already awaiting treatment (Souza, 2014).

Still, most of these centers do not bear all expenses of infertility treatment and/or do not offer the assisted reproduction techniques at all levels of complexity. Even some couples starting treatment at the public healthcare system (SUS) have to pay for essential laboratory tests, afford the medication used, and they have restricted number of induction cycles allowed (Souza, 2014). Only in five human reproduction centers in Brazil patients do not need to bear the cost of the stimulus protocol and they do not have limited number of cycles to be performed (Makuch *et al*., 2011). Our Infertility and Fetal Medicine Center at the Alvaro Alvim Teaching Hospital, is one of those centers where treatment is fully paid by a public program of municipal healthcare, although some supplemental tests are not included.

Brazilian studies (Souza, 2014) reported that another important factor of human reproduction in the country is that not all couples have access to assisted reproductive services by the public healthcare system, because the centers themselves establish inclusion and exclusion criteria, or because most of them are located in big cities, providing services only to couples living in those regions, restricting access to many who live in remote areas.

Data from the Demographic and Health Survey - 2006 (PNDS, 2009) reported that in the last five years preceding the publication, 46% of births derived from unplanned conceptions and other 18% from unwanted conceptions. Although it corroborates events in countries where assisted reproduction is part of a public assistance program, in addition to the clear need for attention to these couples that have their reproductive rights hampered, these data show a clear need for implementation and intensification of public actions regarding reproductive planning.

According to the Family Planning Act 1996 (Act 9263), Article 226, paragraph 2, one can understand that "family planning is a set of fertility regulation actions that ensure equal rights under the constitution, limiting or increasing the progeny of women, men or the couple;" and among the specified actions listed on paragraph 3, assistance in contraception and conception, making family planning a set of global and comprehensive framework of actions. Thus, according to the commitments established by the Constitution, based on the principles of the SUS, it would be up to the State to provide the treatment of both low and high complexity and bear the high cost of assisted reproduction (Garcia *et al*., 2012).

The term reproductive planning seems to be more illuminating than the usual "family planning" that can be confused with acts of "birth control". According to the National Demographic and Healthcare of Children and Women17, an effective reproductive planning policy offering clarification on contraceptive and conceptive methods, seems to be the best way to improve reproductive assistance and change this situation.

## CONCLUSION

The profile of couples seeking the public healthcare system for infertility treatment is: low-income, low education, having more children and the tubal factor, due to tubal ligation, is the major cause of infertility. The cost of treatment is still a limiting factor for most couples and effective public healthcare policies could minimize the problem, since it is still outdated and not comprehensive in this field.

The public healthcare system cannot meet all the demand, and infertility treatment is restricted to a minority who can afford the services on the private healthcare network. Reproductive rights and their enforcement depend on access to reproductive planning services, for both contraception and conception care. Public policy should be adopted for the State to guarantee reproductive rights at all levels of complexity.
